# Reviews of Medical Books

**Published:** 1856-10

**Authors:** 


*Reviews of Medical Books.*—A short time since, one of the western medical journals acknowledged the receipt of a valuable and laborious work, by a review of precisely five lines in length. All that was said was laudatory in the extreme. How such a notice pleases a publisher, may be guessed from the following extract from a letter written by the distinguished publishing house which forwarded the book for notice.

"Those five lines of unmeaning praise are the type of numerous others, and these very journalists, who ignore the duty they owe to American literature, are the foremost in deploring the want of independence among our authors and publishers. If a book is worthless let the fact be candidly stated; but an elaborate work by a man of position, certainly deserves an intelligent analysis and exposition of its characteristics, good or bad. We feel as lively an interest, (and a much more practical one) in advancing the character of our native medical literature as any of those who find in it a text of never-failing freshness; but we would ask you whether this careless smothering is not well fitted to dampen the ardor of bookseller as well as author?"

The question is well put, and though not designed for our latitude, we feel, to a certain extent, the sharp rebuke it contains. We have, however, never felt that duty, either to publisher or public, was fully discharged in the mere acknowledgment of the reception of a book. This acknowledgment amounts to an advertisement. The readers of a journal prefer to have their advertisements in a body on the cover, and, when a new book is published, they wish to know the opinion of one who has read it, whether it is worth while for them to purchase it. In the large space devoted to a full review, the editor is apt to forget that he is serving, not the publisher, but his readers.

We do not know of any journal—ourselves not excepted—which is not more or less open to the charge so well urged by these publishers. At any rate, let journalists do *something* in the way of reviews of American books. If they need abusing, it is a pleasant and profitable recreation to administer the necessary castigation. We all have a warm, cordial feeling in showing up a writer's infirmities. Let people talk as they may about "unpleasant duty," and so forth, detraction is one of the most delightful of vices. But when one of our countrymen, whether of acknowledged fame and reputation, or struggling to obtain a place among the *nomina digna*, has sent out to the world the fruit of weary labors, carried through a series of years, rich, like the pomegranite, with seeds of future successes, let us hold out to him the American hand, warm and friendly, and render an honor which will be felt by him as not merely kind, but intelligent and appreciative. When we have done this, and so given some encouragement to American authors and publishers, then we may go in conscientiously to root out the worthless portion of the European literature republished here.

